# *Helicobacter pylori*-Induced Progranulin Promotes the Progression of the Gastric Epithelial Cell Cycle by Regulating CDK4

**DOI:** 10.4014/jmb.2203.03053

**Published:** 2022-06-20

**Authors:** Zongjiao Ren, Jiayi Li, Xianhong Du, Wenjing Shi, Fulai Guan, Xiaochen Wang, Linjing Wang, Hongyan Wang

**Affiliations:** 1 Department of Pathogenic Microbiology, Basic Medical College, Weifang Medical University, Weifang 261053, Shandong, P.R. China; 2 Key Lab for Immunology in Universities of Shandong Province, Basic Medical College, Weifang Medical University, Weifang 261053, Shandong, P.R. China; 3 Department of Gynecology, Weifang Medical University Affiliated Hospital, Weifang 261000, Shandong, P.R. China; 4 Laboratory of Morphology, Weifang Medical University, Weifang 261053, Shandong, P.R. China; 5 Clinical Medical College, Weifang Medical University, Weifang 261053, Shandong, P.R. China

**Keywords:** *Helicobacter pylori*, PGRN, CDK4, gastric epithelial cells, cell cycle

## Abstract

*Helicobacter pylori*, a group 1 carcinogen, colonizes the stomach and affects the development of stomach diseases. Progranulin (PGRN) is an autocrine growth factor that regulates multiple cellular processes and plays a tumorigenic role in many tissues. Nevertheless, the mechanism of action of PGRN in gastric cancer caused by *H. pylori* infection remains unclear. Here, we investigated the role of PGRN in cell cycle progression and the cell proliferation induced by *H. pylori* infection. We found that the increased PGRN was positively associated with CDK4 expression in gastric cancer tissue. PGRN was upregulated by *H. pylori* infection, thereby promoting cell proliferation, and that enhanced level of proliferation was reduced by PGRN inhibitor. CDK4, a target gene of PGRN, is a cyclin-dependent kinase that binds to cyclin D to promote cell cycle progression, which was upregulated by *H. pylori* infection. We also showed that knockdown of CDK4 reduced the higher cell cycle progression caused by upregulated PGRN. Moreover, when the PI3K/Akt signaling pathway (which is promoted by PGRN) was blocked, the upregulation of CDK4 mediated by PGRN was reduced. These results reveal the potential mechanism by which PGRN plays a major role through CDK4 in the pathological mechanism of *H. pylori* infection.

## Introduction

Gastric cancer is the fourth-leading cause of cancer mortality, with over 700,000 deaths each year. The incidence of gastric cancer is highest in Eastern Europe, Eastern Asia, and South America [1, 2]. Due to a low rate of early detection, most gastric cancer patients are generally diagnosed at a late stage, and the overall five-year survival rate is about 20% [3, 4]. *H. pylori* infection is a key pathogenic cause for gastric carcinoma and it has been recognized as a group 1 carcinogen by the WHO [5]. *H. pylori* infects more than half of the global population, and nearly all noncardiac gastric cancers are attributed to this bacterium [6, 7]. A variety of virulence factors produced by *H. pylori* can cause a chronic inflammatory response in the gastric mucosa, which then develops into gastric or duodenal ulcers, atrophic gastritis, gastric cancer, or gastric mucosa-associated lymphoid tissue lymphoma [8, 9]. Although major progress has been made in the diagnosis and treatment of *H. pylori* in recent years, the pathogenesis of *H. pylori*-induced gastric cancer is still unclear.

The cell cycle is a complicated and elaborate regulatory process influenced by multiple factors both inside and outside the cell, where cells generate two daughter cells through a series of replication, division, and growth events under regulating multiple cyclins. Among them, key cycle transitions are driven by different cyclin-dependent kinases (CDKs) and their activated cyclin subunits [10]. CDKs related to cell cycle interphase activation in mammals mainly include CDK4 and CDK6 in G_1_ phase and CDK2 near the beginning of S phase [11]. The CDK4/6 binds to cyclin D and drives the cell-cycle transition from G_1_ to S by phosphorylates retinoblastoma protein (RB)[12]. CDK2 is a core cell-cycle regulator that facilitates the transition from S to G_2_ in the late G_1_ stage by binding to cyclins E and A, and continuing to phosphorylate RB and release E2F transcription factors (E2Fs) [13]. Indeed, the cell-cycle checkpoints of cancer are often associated with DNA damage and genetic defects [14, 15]. Disorders in the cell cycle disrupt normal mitosis, often causing uncontrolled proliferation, leading to cancer development [16]. In the early G_1_-S checkpoint of rat esophageal cancer caused by zinc deficiency, the expression of cyclin D1, CDK4, and RB increases, the p16INK4a cycle D1/cycle-dependent kinase 4 RB pathway is dysregulated, and this is closely associated with cell proliferation [17]. Therefore, the study of cell cycle changes is an important entry point to study cell proliferation.

Progranulin (PGRN), is a growth factor consisting of 593 amino-acid residues, and is also called granulin-epithelin precursor, proepithelin, acroglanin, or GP88. PGRN plays a crucial role in miscellaneous physiological processes involving cell development, cell cycle progression, wound healing, repair and formation of blood vessels and tissues, inflammation, and the growth of bone and cartilage [18-26]. As an important regulatory factor in tumors, PGRN is strongly expressed in various tumors, including cervical cancer, prostate cancer, bladder cancer, colorectal cancer, and lymphoma, and is associated with overall survival [27-31]. Studies have shown that inhibition of PGRN can inhibit tumor growth. For example, PGRN repression inhibits the proliferation of hematopoietic cancer cells [32]. Blocking PGRN can suppress the proliferation and migration of triple-negative breast cancer cells [18]. Furthermore, studies have demonstrated that PGRN regulates the expression of tumor-associated macrophage PD-1, promotes CD8+ T cell rejection, and induces breast cancer immune escape [33]. Thus, the high expression of PGRN is closely associated with the progression of malignancies [34]. However, the mechanism by which PGRN induces cellular responses in *H. pylori* infected cells remains unclear.

Studies have shown that the virulence factor Cag A of *H. pylori* can promote cell proliferation by affecting cell cycle progression [35]. We have previously reported that PGRN is upregulated by *H. pylori* through the p38MAPK and MEK1/2 signaling pathways, and then promotes the migration and proliferation of gastric epithelial cells [36]. However, the role of PGRN in *H. pylori*-induced cell cycle progression remains unclear, and the potential mechanisms are still to be illustrated. In this research, we found not only that PGRN and CDK4 were both overexpressed in gastric cancer, but there was also a positive correlation between them. The upregulation of PGRN induced by *H. pylori* increased the cell cycle progression and the proliferation of gastric epithelial cells. As a target gene of PGRN, CDK4 participated in the regulation of the cell cycle. This study is the first to investigate the function and mechanism of PGRN and CDK4 in cell cycle progression and proliferation induced by *H. pylori* in assays performed in vitro.

## Materials and Methods

### Tissue Samples

One hundred gastric cancer tissue and adjacent normal tissue samples were provided by Weifang Peoplés Hospital and the Affiliated Hospital of Weifang Medical University through gastroscopy and gastric cancer surgery, respectively. The age, gender, and relevant clinical data of all subjects were collected as approved by the Ethics Committee of Weifang Medical University (2022YX045). There was no statistical difference in the tissue sources of each group in age, gender, TNM stage, and other related indicators.

### Cell Culture and Reagents

BGC-823 gastric cancer cells were grown in RPMI 1640 (Gibco, USA) supplemented with 10% newborn bovine serum (Gibco) in a CO_2_ incubator at 37°C containing 5% CO_2_. The PI3K/Akt inhibitor LY294002, the nuclear factor-κB (NF-κB) inhibitor BAY11-7082, and the MAPK inhibitor UO126 were from Cell Signaling Technology (USA). The three signal pathway inhibitors were dissolved in dimethyl sulfoxide (DMSO, China) solution.

### H. pylori Culture

H. pylori strain 26695 was maintained in our laboratory. Bacteria were incubated in Brucella broth with 5% fetal bovine serum at 37°C under microaerophilic conditions containing 10% CO_2_, 5% O2 and 85% N_2_. Depending on the experimental requirements, BGC-823 cells were infected at different multiplicity of infection (MOI) of *H. pylori*.

### Lentiviral Vector Construction and Transfection

PGRN knockdown lentivirus pLKO.1-PGRN shRNA-GFP vector and PGRN overexpression lentivirus Plenti6/V5-PGRN vector, and the corresponding negative control vector were successfully constructed and preserved in the laboratory. Lentiviruses CDK4-RNAi-13, CDK4-RNAi-14, CDK4-RNAi-15, and their control vector were purchased from Shanghai Genechem Co., Ltd. (China). BGC-823 cells were seeded in 6-well plates, and the virus was infected when the cell density reached ~70%. The PGRN knockdown group (represented by SI) and the control group (empty vector, represented by NS), the PGRN-overexpressing group (represented by PGRN) and the control group (empty vector, represented by GFP) were transfected with Lipofectamine 2000 (Invitrogen, USA). All experiments were carried out in triplicate according to the manufacturer's instructions.

### RNA Extraction and Quantitative Real-Time PCR (qPCR)

Based on the manufacturer's instructions, total RNA was extracted with TRIzol (Invitrogen). cDNA was synthesized from 2 μg of extracted RNA using a ReverTra Ace qPCR RT Kit (Toyobo, Japan). The SYBR Green Pro Taq HS Premix qPCR Kit (AG, China) and the ABI 7900HT System (ABI, USA) were used for qPCR of cDNA expression. β-Actin was utilized as the normalization control. The relative expression fold changes of mRNA were calculated using the 2^-ΔΔCt^ comparative threshold cycle method [37]. The primer sequences used were as follows: PGRN: forward-5’-GGACAGTACTGAAGACTCTG-3’, reverse-5’-GGATGGCAGCTTGTAATGTG-3’; CDK4: forward-5’-GGGCCGAGAGGACAGAATGG-3’, reverse-5’-GCTGTTCTAATCACCAGGGTAGGCC-3’; β-actin: forward-5’-AGTTGCGTTACACCCTTTCTTG-3’, reverse-5’-CACCTTCACCGTTCCAGTTTT-3’.

### Western Blot Analysis

Gastric cancer cell proteins were collected using RIPA lysis buffer with PMSF protease inhibitor (Solarbio, China). The total protein was separated by SDS-PAGE and then transferred to a PVDF membrane. The PVDF membrane was blocked with 5% skim milk in TBST buffer for 1 h at room temperature and then incubated with primary antibody at 4°C overnight. Following that, it was incubated with HRP-linked anti-mouse IgG (1:2000, #7076, Cell Signaling Technology, USA) or anti-rabbit IgG (1:2000, #7074, Cell Signaling Technology) at room temperature for 1 h. The bands were visualized by a chemiluminescence ECL detection system (EMD Millipore, USA). The primary antibodies used were: anti-PGRN (1:200, sc-377036, Santa Cruz, USA), anti-CDK4 (1:1000, #12790, Cell Signaling Technology), anti-β-actin (1:1000, sc-47778, Santa Cruz), anti-Akt (1:1000, 4691S, Cell Signaling Technology), and anti-p-Akt (1:2000, #4060S, Cell Signaling Technology).

### Immunohistochemical Analysis

Paraffin-embedded tissue sections were dewaxed and dehydrated using xylene and ethanol, respectively. After antigen retrieval, the samples were blocked with goat serum working solution (ZSGB Biotech, China). They were then incubated at 4°C overnight after adding mouse anti-PGRN antibody (1:100, sc-377036, Santa Cruz) or rabbit anti-CDK4 antibody (1:800, #12790, Cell Signaling Technology) according to the instructions. According to the difference in the primary antibody, the secondary antibody biotin-labeled goat anti-mouse IgG (1:700, ZSGB Biotech) or goat anti-rabbit IgG (1:700, ZSGB Biotech) was applied, followed by drop-wise HRP-conjugated Streptavidin working solution (ZSGB Biotech). Then, DAB chromogenic solution (ZSGB Biotech) was used for color development followed by hematoxylin staining for 1 min and 1% hydrochloric acid alcohol for color separation for 3–4 s. Then, 0.2% ammonia and neutral gum (Biosharp, China) were used for sealing after dehydration. Images were analyzed with Image Pro Plus 6.0 (Media Cybernetics, USA) software.

### Flow Cytometry

The cells were cultured to a density of 90%, collected, washed 3 times with PBS, and fixed in 1 ml of 70% low-temperature ethanol at 4°C overnight. After washing with PBS, cells were stained with 0.5 ml propidium iodide (25 μl propidium iodide, 10 μl RNaseA, Beyotime, China). Following that, the cells were re-suspended and bathed in water at 37°C for 30 min. FACSVerse flow cytometry (BD, USA) was used to assess the cell cycle.

### Colony Formation Assay

The cells were or were not infected with *H. pylori* for 3 h, then 300 cells were counted, seeded in a 6-well plate, and cultured at 37°C and 5% CO_2_ for 14 days. After fixation with methanol, they were stained with Giemsa dye (Solarbio), counted, and photographed.

### Statistical Analysis

All data are indicated as the mean ± SD and were statistically analyzed using GraphPad Prism 8.0 (GraphPad Software Inc., USA). Comparison between groups was tested by paired *t*-test. One-way ANOVA was used to determine the differences in multiple comparison. Correlations of protein expression were done using the Spearman rank correlation test. *p*<0.05 represents statistical significance.

## Results

### PGRN and CDK4 Are Overexpressed in Gastric Cancer Tissues

**Fig. 1:**
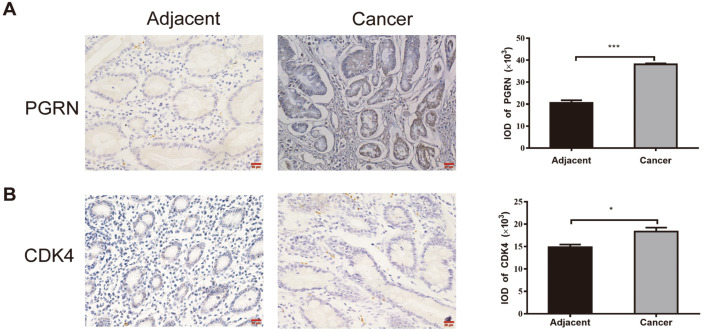
Differences of PGRN and CDK4 protein expression between gastric cancer tissue and adjacent normal tissue. (A, B) Expression levels of PGRN (**A**) and CDK4 (**B**) in both gastric cancer and adjacent normal tissues as measured by immunohistochemistry. The results represent the mean ± SD of three independent experiments. *p < 0.05, **p < 0.01, ***p < 0.001, paired *t*-test. All data are mean values of three biological replicates.

The expression levels of PGRN and CDK4 in adjacent normal tissue and gastric cancer were assessed by immunohistochemical staining. Compared with adjacent normal tissue, PGRN (Fig. 1A) and CDK4 (Fig. 1B) expressed significantly higher in gastric cancer tissue. By analyzing the correlation between PGRN and CDK4, we found that PGRN was positively associated with CDK4 in gastric cancer, and the correlation coefficient r was 0.452 (Table 1).

### *H. pylori* Infection Regulated the Proliferation and Cycle Progression of Gastric Epithelial Cells

**Fig. 2:**
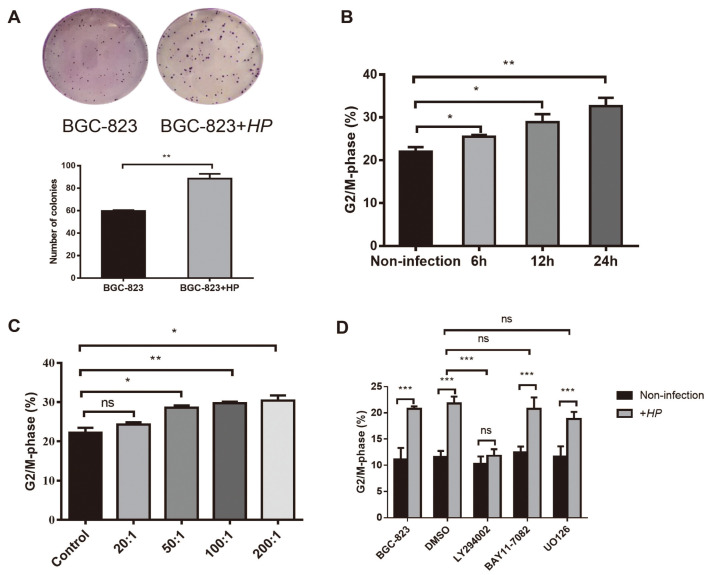
*H. pylori* infection promotes cell cycle progression and cell proliferation. (**A**) BGC-823 cells cocultured with *H. pylori* at a multiplicity of infection (MOI) 50:1 for 3 h, and their clonogenic potential were then assessed. (**B**) Flow cytometry results of BGC-823 cells infected with *H. pylori* 26695 at a MOI of 50:1 for 6, 12, and 24 h. (**C**) Flow cytometric results of *H. pylori* 26695 infected BGC-823 cells at different MOIs (10:1, 20:1, 50:1, 100:1, and 200:1). (**D**) Flow cytometry results of BGC-823 cells pre-treated with BAY11-7082 (5 μM), LY294002 (10 μM) and UO126 (10 μM) for 2 h before incubation with or without *H. pylori* at a MOI of 50:1 for 12 h. The results represent the mean ± SD of three independent experiments. ns, not significant, HP, *Helicobacter pylori*, *p < 0.05, **p < 0.01, ***p < 0.001.

To evaluate whether *H. pylori* infection influenced the proliferation of gastric epithelial cells, we co-incubated BGC-823 cells with *H. pylori* at an MOI of 50:1. The results showed that, compared with the non-infected group, the proliferative capacity of BGC-823 cells was substantially increased after infection with *H. pylori* (Fig. 2A), indicating that *H. pylori* infection increases cell proliferation.

To explore the mechanisms by which *H. pylori* promotes cell growth, we analyzed the effect of *H. pylori* infection on the cell cycle. BGC-823 cells were infected with *H. pylori* at an MOI of 50:1 for 6, 12, and 24 h. Flow cytometry analysis showed that, compared with uninfected cells at each time point, cells entering G_2_/M phase increased significantly after *H. pylori* infection, and with the prolongation of infection time, the proportion of cells entering G_2_/M phase gradually increased (Fig. 2B). Next, BGC-823 cells were infected with *H. pylori* at varying MOIs of 10:1, 20:1, 50:1, 100:1, and 200:1 for 12 h. Flow cytometry analysis demonstrated that with the increase of MOI, the proportion of cells entering G_2_/M phase gradually increased (Fig. 2C). These results suggested that *H. pylori* infection regulates gastric epithelial cell cycles in a time- and dose-dependent manner.

*H. pylori* was found to activate a set of main signaling molecules that include NF-κB, PI3K/Akt, and mitogen-activated protein kinases (MAPKs). To clarify the signaling pathways regulating the *H. pylori*-induced cell cycle, three signal molecule inhibitors were added to BGC-823 cells 2 h prior to *H. pylori* infection at an MOI of 50:1. Flow cytometry results indicated that only PI3K/Akt inhibitor LY294002 (10 μM) was capable of inhibiting a higher proportion of cells entering G_2_/M significantly stimulated by *H. pylori* and decreased the cell cycle to basal level, and there was no significant difference in the cell cycle using NF-κB inhibitor BAY11-7082 (5 μM) and MAPK inhibitors UO126 (10 μM) (Fig. 2D). Therefore, *H. pylori* infection may regulate the cell cycle via the PI3K/Akt signaling pathway.

### PGRN Promotes *H. pylori*-Induced Gastric Epithelial Cell Cycle Progression and Cell Proliferation

**Fig. 3:**
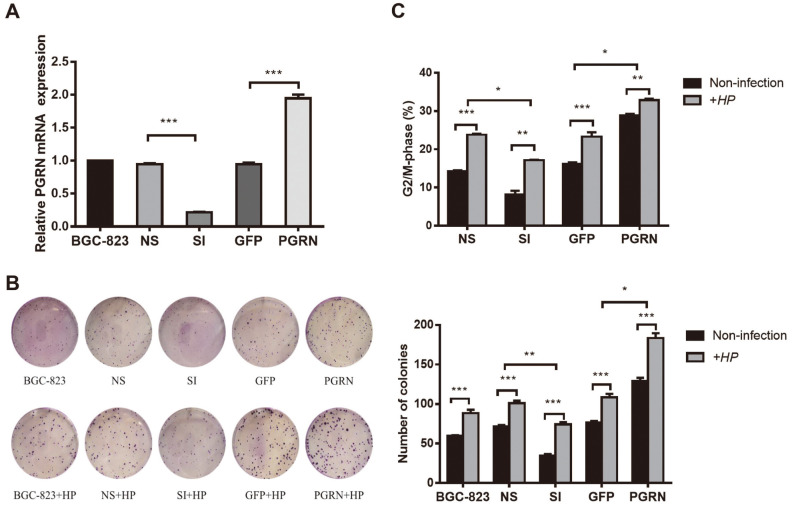
PGRN promotes cell cycle progression and cell proliferation in gastric cancer cells with or without *H. pylori* infection. (**A**) qPCR analysis of the expression of PGRN after transfection with pLKO.1-PGRN shRNA-GFP and Plenti6/V5-PGRN lentivirus. (**B, C**) Colony formation assays (**B**) and cell cycle assays (**C**) of BGC-823 cells transfected with pLKO.1-PGRN shRNA-GFP and Plenti6/V5-PGRN and their negative control lentivirus and cocultured with *H. pylori* at a MOI of 50: 1 for 3 h. The results represent the mean ± SD of three independent experiments. SI, PGRN knockdown group, NS, the control group of PGRN knockdown, GFP, the control group of PGRN overexpressing, HP, *Helicobacter pylori*, *p < 0.05, **p < 0.01, ***p < 0.001.

Our previous studies have demonstrated that *H. pylori* increases PGRN expression via the p38MAPK and MEK1/2 pathways in gastric epithelial cells. Therefore, we sought to examine the role of PGRN in the gastric epithelial cell cycle progression and the cell proliferation induced by *H. pylori* infection. We knocked down and overexpressed PGRN in BGC-823 cells by lentivirus pLKO.1-PGRN shRNA-GFP (represented by SI), the control group (empty vector, represented by NS), the PGRN overexpressing lentivirus Plenti6/V5-PGRN (represented by PGRN), and the control group (empty vector, represented by GFP). The qPCR verified the effectiveness of lentivirus infection (Fig. 3A). Then, we co-incubated BGC-823 cells with *H. pylori* at an MOI of 50:1. Colony formation assay showed that repression of PGRN markedly reduced the foci numbers as well as sizes but overexpression of PGRN led to a significant increase. *H. pylori* infection could obviously increase the colony formation, but downregulation of PGRN nearly decreased the proliferative ability promoted by *H. pylori* infection, while overexpression of PGRN significantly enhanced the proliferation induced by *H. pylori* (Fig. 3B). Consistent with these results, the proportion of cells progressing to G_2_/M after PGRN knockdown was markedly less than that in the control group, but the proportion was markedly higher in PGRN overexpression. *H. pylori* infection could accelerate cell cycle progression to G_2_/M, but knockdown by PGRN almost reduced these activities induced by *H. pylori*, while overexpression of PGRN enhanced these activities (Fig. 3C). These results indicated that upregulating PGRN is associated with cell cycle progression and cell proliferation induced by *H. pylori* infection.

### *H. pylori* Increases Expression of CDK4 to Promote the Cell Cycle through the Upregulation of PGRN

**Fig. 4:**
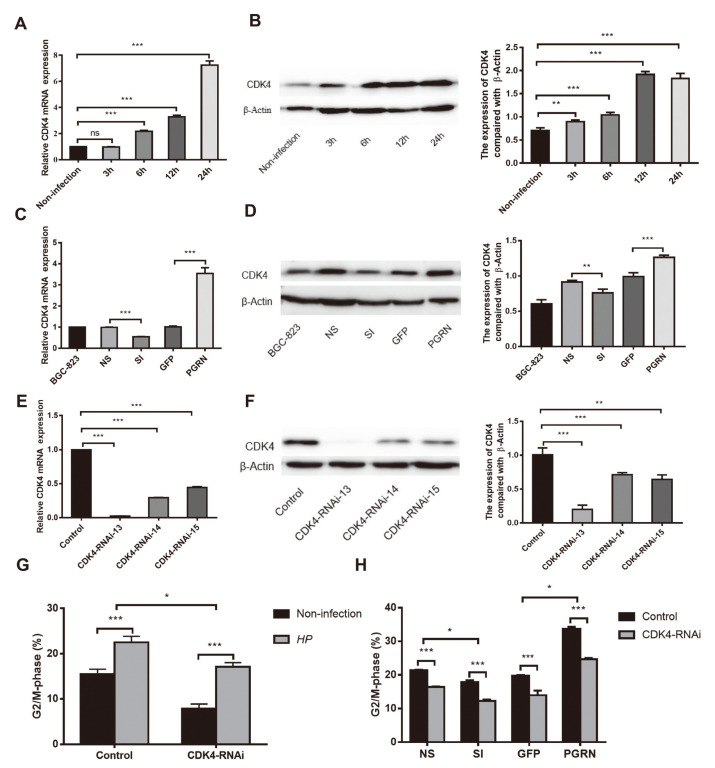
PGRN positively regulates CDK4 to promote cell cycle progression. (**A, B**) qPCR and western blot analysis of the expression of CDK4 in BGC-823 cells infected with *H. pylori* 26695 at a MOI of 100: 1. (**C, D**) qPCR and western blot analysis of the expression of CDK4 after transfection with pLKO.1-PGRN shRNA-GFP and Plenti6/V5-PGRN lentivirus. (**E, F**) CDK4 expression after transfection with CDK4-RNAi-13, CDK4-RNAi-14, CDK4-RNAi-15 of CDK4-knockdown lentivirus. (**G**) Flow cytometry analysis of the cell cycle changes of CDK4 knockdown and coculture with *H. pylori* in 50:1 MOI in BGC-823 cells. (**H**) Flow cytometry analysis of the cell cycle changes of CDK4 knockdown cell lines infected with PGRN-knockdown /overexpressed lentivirus and cocultured with *H. pylori* at a MOI of 50: 1. The results represent the mean ± SD of three independent experiments. SI, PGRN knockdown group, NS, the control group of PGRN knockdown, GFP, the control group of PGRN overexpressing, ns, not significant, HP, *Helicobacter pylori*, *p < 0.05, **p < 0.01, ***p < 0.001.

We have confirmed that the expression of PGRN and CDK4 was both increased and positively correlated in gastric cancer, but it was not clear whether the upregulated PGRN regulated the cell cycle via CDK4. BGC-823 cells were cocultured with *H. pylori* at an MOI of 100:1. Compared with the non-infected group, CDK4 mRNA and protein expression were both apparently upregulated after *H. pylori* infection. Furthermore, CDK4 expression was elevated in a time-dependent manner (Figs. 4A and 4B).

**Fig. 5:**
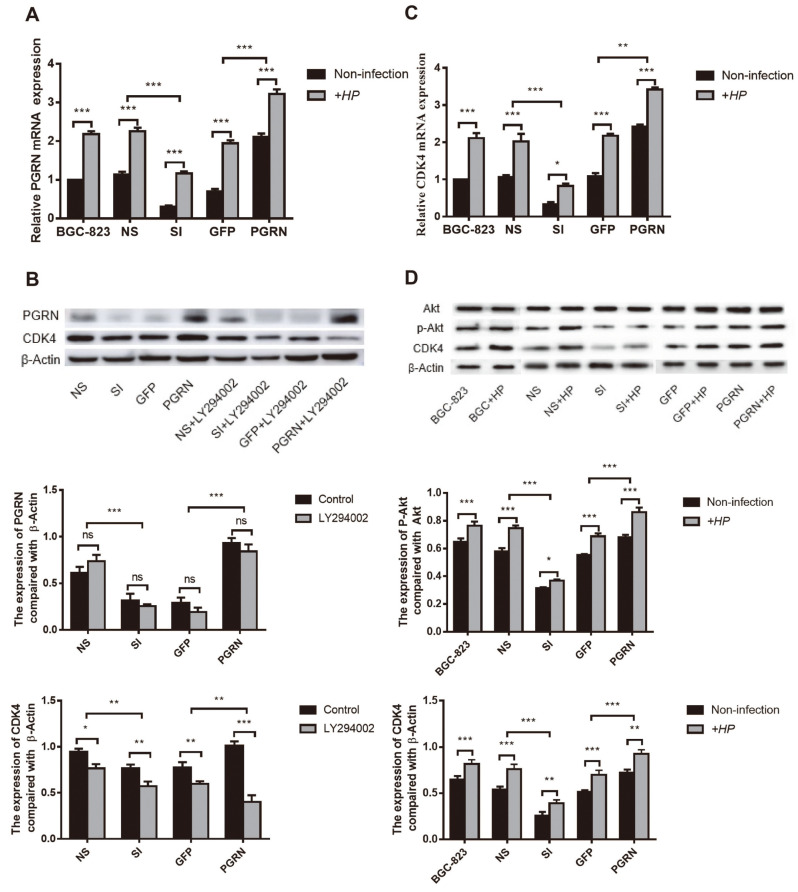
PGRN regulates CDK4 through the PI3K/Akt signaling pathway and promotes progression of the gastric mucosal epithelial cell cycle. (**A**) qPCR analysis of the expression of PGRN after transfection with pLKO.1-PGRN shRNA-GFP and Plenti6/V5-PGRN lentivirus and cocultured with *H. pylori* at a MOI of 100: 1. (**B**) Western blot analysis of CDK4 protein expression in cells pretreated with a PI3K signal pathway inhibitor (LY294002) for 2 h before transfection with pLKO.1-PGRN shRNA-GFP and Plenti6/V5-PGRN lentivirus. (**C**) qPCR analysis of the expression of CDK4 after transfection with pLKO.1-PGRN shRNA-GFP and Plenti6/V5-PGRN lentivirus and cocultured with *H. pylori* at a MOI of 100: 1. (**D**) Western blot analysis of CDK4, Akt, and p-Akt protein expression in cells transfected with pLKO.1-PGRN shRNA-GFP and Plenti6/V5-PGRN lentivirus and cocultured with *H. pylori* at a MOI of 100: 1. The results represent the mean ± SD of three independent experiments. SI, PGRN knockdown group, NS, the control group of PGRN knockdown, GFP, the control group of PGRN overexpressing, HP, *Helicobacter pylori*, *p < 0.05, **p < 0.01, ***p < 0.001.

To define the role of PGRN on CDK4 expression, we transfected BGC-823 cells with lentivirus pLKO.1-PGRN shRNA-GFP and lentivirus Plenti6/V5-PGRN. Western blot confirmed that the repression of PGRN markedly decreased the expression of CDK4, and overexpression of PGRN apparently promoted the expression of CDK4 (Figs. 4C and 4D). Furthermore, *H. pylori* infection increased CDK4 expression in BGC-823 cells; however, this increased expression was decreased after knockdown of PGRN and upregulated after overexpression of PGRN (Figs. 5C and 5D). These results suggested that *H. pylori* regulated CDK4 expression via PGRN.

To verify the effect of CDK4 on the cell cycle, we transfected BGC-823 cells with lentiviruses CDK4-RNAi-13, CDK4-RNAi-14, and CDK4-RNAi-15. qPCR and western blot showed that CDK4-RNAi-13 successfully inhibited the expression of CDK4, and the inhibition efficiency of CDK4-RNAi-14 and CDK4-RNAi-15 was relatively lower (Figs. 4E and 4F). We transfected BGC-823 cells with CDK4-RNAi-13 and assessed their cycle distribution by flow cytometry. The results indicated that the progression to G_2_/M was significantly reduced after CDK4 repression and the repression of CDK4 could attenuate the higher progression to G_2_/M induced by *H. pylori* (Fig. 4G).

To further verify that PGRN promoted the cell cycle of the gastric epithelial cells via CDK4, BGC-823 cells were co-transfected with CDK4-RNAi-13 and lentivirus pLKO.1-PGRN shRNA-GFP or lentivirus Plenti6/V5-PGRN. Fewer cells progressed to G_2_/M in PGRN and CDK4 both repression groups compared with repression of PGRN or CDK4 alone. The proportion of cells entering G_2_/M after overexpression of PGRN was markedly higher than that in the control group, while co-transfection with CDK4-RNAi-13 reduced this proportion nearly to the baseline (Fig. 4H). These results further indicated that *H. pylori* upregulated CDK4 expression, thereby promoting cell cycle progression via the upregulation of PGRN.

### PGRN Regulates CDK4 via PI3K/Akt Signaling Pathway

We have already demonstrated that *H. pylori* infection may regulate the cell cycle via the PI3K/Akt signaling pathway. To analyze whether PGRN regulates CDK4 expression via the same pathway, we next applied the PI3K signal pathway inhibitor LY294002 to lentivirus pLKO.1-PGRN shRNA-GFP or lentivirus Plenti6/V5-PGRN infected BGC-823 cells. qPCR verified the effectiveness of PGRN knockdown and overexpression (Fig. 5A). LY294002 can markedly repress CDK4 expression stimulated by PGRN (Fig. 5B). To investigate whether PI3K signal pathway participates in the signal transduction process, western blot was used to evaluate that phosphorylation of Akt in response to PGRN knockdown or overexpression and interaction with *H. pylori*. We found that Akt phosphorylation and CDK4 expression decreased in PGRN knockdown cells, while their expression increased in overexpressing PGRN. Infected with *H. pylori*, Akt phosphorylation and CDK4 expression were higher than that without *H. pylori*, while the repression of PGRN could reduce the higher phosphorylation and CDK4 expression, and the overexpression of PGRN could augment them. (Figs. 5C and 5D). This indicated that the increased PGRN induced by *H. pylori* infection regulated CDK4 expression via PI3K/Akt signaling pathway. Consequently, the increased CDK4 promoted gastric epithelial cell cycle progression.

## Discussion

As a multifunctional growth factor, PGRN is involved in cell growth, inflammation regulation, tumorigenesis, and many other important aspects. In a mouse model of PGRN-deficient arthritis, PGRN inhibits the binding of TNF to its receptor and blocks intracellular signaling pathways [38]. PGRN overexpression promotes the secretion of multiple inflammatory factors that contribute to the development of tumors and related diseases [39, 40]. Many studies have indicated that PGRN is overexpressed in various human cancers, for instance, ovarian cancer, colorectal cancer, and gastrointestinal tumors [41-43]. We previously showed that the upregulation of PGRN induced by *H. pylori* accelerates the cell proliferation and migration of gastric epithelial cells [36]. In this report, we showed that PGRN was upregulated by *H. pylori* infection in gastric epithelial cells, thereby stimulating the cell cycle and promoting cell proliferation by increasing the expression of CDK4. Immunohistochemical analysis demonstrated that the expression of PGRN and CDK4 in gastric cancer tissue was higher than that in adjacent normal tissue, and PGRN was positively associated with CDK4 in gastric cancer. As a highly tumorigenic growth factor, overexpression of PGRN in weakly tumorigenic cells significantly promotes tumor growth [44]. Inhibition of PGRN expression in highly tumorigenic mouse cells can reduce tumor formation [45]. This is consistent with our findings. In breast cancer, the tissue level of PGRN predicts the risk of recurrence of ER-positive invasive ductal carcinoma [46]. Monoclonal antibody against PGRN inhibits the growth of hepatocellular carcinoma in nude mice [47]. This shows that PGRN is a feasible target for developing new drugs against certain cancers.

*H. pylori*, as the main pathogenic factor of gastric cancer, has a significantly increased infection rate in premalignant lesions and gastric cancer. Eradication of *H. pylori* reduces the incidence of gastric cancer [48, 49]. Current research has shown that a variety of virulence factors produced by *H. pylori*, for instance, CagA, VacA, HtrA, Baba, Saba, and oipa, can help it attach to gastric epithelial cells, cause the host immune system to release various pro-inflammatory cytokines and chemokines and activate multiple signal pathways, such as the NF-κB, Wnt/β-catenin, and PI3K/Akt/mTOR pathways, which affect cell proliferation and differentiation, and promote the transformation of normal gastric epithelial cells into cancer cells [50, 51]. In this study, we demonstrated that *H. pylori* increased the proliferative activity of gastric epithelial cells in a certain range, and the increased activity was positively correlated with the number of bacteria loaded. In addition, we found that the more proliferative activity induced by *H. pylori* was caused by accelerated cell cycle progression. The cell cycle is an important event associated with development, apoptosis, DNA repair, and tissue regeneration [52-54]. This also confirms that *H. pylori* plays a crucial role in tumorigenesis and metastasis [55]. Normal proliferation of cells is regulated by cell cycle checkpoints, and once the cell cycle checkpoint is defective, cells may proliferate uncontrollably [56, 57]. When cancer occurs, the control of checkpoints often becomes dysfunctional, including abnormal expression of the RB gene and mis-regulation of CDKs, resulting in dysregulated cell cycle activity, causing hyper-proliferation leading to cancer or enabling cell loss [16, 58]. The CDK-cyclin complex regulates the cell cycle process by phosphorylating its substrates, and the cycle process is negatively regulated by cell cycle-dependent kinase inhibitors (CDKIs), which can halt the process by binding inhibition before or after DNA replication in response to DNA damage [50, 59]. CDKIs are divided into INK4 families (including p16^ink4c^, p15^ink4c^, P18^ink4c^, and P19^ink4c^) and CIP/Kip families (p21^cip1^, p27^kip1^, and p57^kip2^) [60]. In a p21^cip1^ and p27^kip1^-deficient mouse model, the tumor growth rate was accelerated [61]. The action of *H. pylori* on the cell cycle may be connected with its regulated CDKs. Ahmed and Li detected that *H. pylori* infection caused DNA damage, increased p53 expression, which induced p21 expression, and bound to the CDK2-cyclin E complex to block the cell cycle in G_1_ phase [62, 63]. However, Sherr and Shirin found that *H. pylori* inhibits the expression of p27^kip1^, which binds to cyclin E and CDK2 and inhibits the transition from G_1_ to S. Similarly, other studies have shown that *H. pylori* promotes the expression of cyclin D1, which causes activation of CDK4 and CDK6, initiates the inactivation of the phosphorylation-dependent RB tumor suppressor protein and the release of transcription factor E2F, and shortens G_1_ phase and increases the proliferation rate [64, 65]. Some studies have suggested that *H. pylori* infection caused cell cycle arrest [66-69]. In this study, we found that *H. pylori* accelerated the cell cycle process from G_1_ to G_2_/M in a time- and dose-dependent manner, clarifying the promoting action of *H. pylori* on the cell cycle. This effect may be related to the time in coculture and the dose of *H. pylori*. Therefore, *H. pylori* may affect the proliferation of cancer cells by disrupting the balance of each stage of the cell cycle by affecting the expression of proteins in each phase of the cell cycle.

Recent studies reported that loss-of-function of PGRN caused the accumulation of TDP-43 protein to inhibit CDK6 expression, and then abnormally activated the Wnt5a signal and showed cell cycle disorder [70, 71]. To evaluate the function of PGRN in the cell cycle induced by *H. pylori* infection, cell cycle distribution was analyzed in gastric epithelial cells. We found that *H. pylori* infection promoted progression to G_2_/M, but knockdown of PGRN reduced these activities promoted by *H. pylori* infection, while PGRN overexpression enhanced these activities. This indicated that the cell cycle-promoting effects induced by *H. pylori* infection may be mediated through PGRN. To further understand the molecular mechanisms of PGRN regulating cell cycle progression, we turned our attention to CDK4, which is positively correlated with PGRN expression in gastric cancer. It has been reported that the synergy of PI3K and CDK4/6 inhibitors increases apoptosis and cell cycle arrest in triple-negative breast cancer cells, and that tumor immunogenicity is enhanced [72]. CDK4/6 inhibitor inhibits tumor growth in xenograft mouse model [73]. Here, we demonstrated that CDK4 is the downstream target of PGRN. Knockdown of PGRN significantly inhibited CDK4 expression, and overexpression of PGRN markedly promoted CDK4 expression. Moreover, we found that CDK4 expression was apparently upregulated in gastric epithelial cells after *H. pylori* infection. Repression of PGRN inhibited the higher expression of CDK4 promoted by *H. pylori*, while overexpression of PGRN further promoted the expression of CDK4, indicating that *H. pylori* increased CDK4 expression through PGRN. Repression of CDK4 could also decrease the cell cycle process induced by *H. pylori* infection. Meanwhile, knockdown of CDK4 expression inhibited the cell cycle progression promoted by PGRN overexpression. These findings demonstrated that *H. pylori* upregulated CDK4 expression to promote cell cycle progression via the upregulation of PGRN.

The PI3K/Akt, NF-κB, and MEK/ERK signaling pathways are important pathways that participate in the process by which PGRN regulates tumor growth [74, 75]. Here, we cocultured cells with various signal pathway inhibitors and found that *H. pylori* regulated the cell cycle via the PI3K/Akt signal pathway. Furthermore, we cocultured PI3K/Akt signaling pathway inhibitors with PGRN-overexpressing cells and found that this inhibitor reduced the expression of CDK4. To determine whether PI3K signal pathway participated in the signal transduction process, the phosphorylation of Akt was detected. The Akt was activated by *H. pylori* infection, and inhibition of PGRN reduced the higher activation, while overexpression of PGRN increased this level. These data showed that the enhanced expression of PGRN stimulated by *H. pylori* activated the PI3K signaling pathway, thereby increasing the expression of CDK4, accelerating the entry of cells into G_2_/M phase, which increased the proliferation of gastric epithelial cells and promoted tumorigenesis. Additionally, other studies have suggested that the growth of mucosal epithelial cells after *H. pylori* colonization may be mediated by a gastrin-dependent mechanism [76]. This also provides a novel approach for extensive exploration of its mechanisms in the future.

In conclusion, our study demonstrated that infection of gastric epithelial cells by *H. pylori* led to increased PGRN expression, which regulated the expression of CDK4 by activating the PI3K/Akt signal pathway. The increased CDK4 then regulated the cell cycle and promoted cell proliferation. This process not only provides a new direction for exploring the carcinogenic pathway of *H. pylori*, but also provides a new potential target for the early detection of and therapy for gastric cancer.

## Figures and Tables

**Table 1:** Correlations between PGRN and CDK4 in gastric cancer and adjacent normal tissues analyzed by linear regression.

	IOD(×10^3^)	r	P
PGRN	38.095 ± 1.60	0.452	0.023
CDK4	18.342 ± 0.84		
